# Khat use in people living with HIV: a facility-based cross-sectional survey from South West Ethiopia

**DOI:** 10.1186/s12888-015-0446-5

**Published:** 2015-04-03

**Authors:** Matiwos Soboka, Markos Tesfaye, Garumma Tolu Feyissa, Charlotte Hanlon

**Affiliations:** 1 Department of Psychiatry, College of Public Health and Medical Sciences, Jimma University, Jimma, Ethiopia; 2 Department of Health Education and Behavioral Science, College of Public Health and Medical Sciences, Jimma University, Jimma, Ethiopia; 3 Department of Psychiatry, School of Medicine, College of Health Sciences, Addis Ababa University, Addis Ababa, Ethiopia; 4 Health Services and Population Research Department, Centre for Global Mental Health, King’s College London, London, UK

**Keywords:** Khat use, HIV, Mental distress, Ethiopia, ‘ART adherence’, Substance use disorder, Sub-Saharan Africa

## Abstract

**Background:**

Khat is an evergreen plant with leaves containing the amphetamine-like compounds cathinone and cathine. Many people in the Horn of Africa use khat on a regular basis. Adverse health and social consequences of khat use have been described but little is known about the use of khat in people living with Human Immunodeficiency Virus (PLHIV) in Ethiopia. This study aimed to assess the prevalence of khat use and factors associated with khat use among PLHIV who are in contact with HIV services in a hospital in south-west Ethiopia.

**Methods:**

A cross-sectional study was conducted among 389 PLHIV who attended HIV services at Jimma University Specialized Hospital in September 2012. A structured questionnaire, translated into the local languages, was used to ask about the frequency of khat use and potential risk factors and consequences of khat use in this patient group. Logistic regression analysis was used for bivariate and multivariable analysis.

**Results:**

The overall prevalence of current khat use among people living with HIV was 23.0%. The prevalence was 18.3% in females and 33.6% in males. Christians were less likely to use khat when compared to Muslims (adjusted Odds Ratio (aOR) 0.26, 95% CI = 0.13, 0.55). There was a positive association between khat use and mental distress (aOR 1.84, 95% CI = 1.01, 3.36), smoking cigarettes (aOR 21.21, 95% CI = 7.19, 62.51), alcohol use disorders (aOR 2.16, 95% CI = 1.10, 4.21), CD4 count ≤200 cells/mm^3^ (aOR 3.46, 95% CI = 1.60, 7.50) and missing at least one dose of antiretroviral medication in the preceding month (ART) (aOR 4.2, 95% CI = 1.80, 5.75).

**Conclusion:**

In this study there was a high prevalence of khat use among people living with HIV which was associated with poorer adherence to ART. There is a need to adapt and evaluate feasible and acceptable interventions to reduce khat use in people living with HIV.

**Electronic supplementary material:**

The online version of this article (doi:10.1186/s12888-015-0446-5) contains supplementary material, which is available to authorized users.

## Background

Khat is a plant with psychoactive properties which is cultivated and used predominantly in East Africa and the Arabian Peninsula [1-3]. People report that they use khat, amongst other reasons, to increase social cohesion or reduce unpleasant emotions [2]. Khat contains the active compounds of cathinone and cathine. Khat users experience a sense of increased energy levels, increased alertness and ability to concentrate, improvement in self-esteem and an increase in libido [4,5]. Additionally, some khat users may experience anxiety, tension, restlessness, hypnogogic hallucinations, hypomania and aggressive behavior or even psychosis [6,7]. Chronic khat use has been said to lead to impairment of mental health, possibly contributing to personality disorders [8,9]. Moreover, khat use is associated with dental problems, gastritis, constipation, oral and esophageal cancer [10] and has vasoconstrictor properties [11] that may lead to hypertension and increased incidence of acute myocardial infarction [12,13].

The prevalence of khat use in Ethiopia varies across populations. In khat producing areas of Ethiopia, the prevalence of khat use is high, varying from 30.6% to 50.0% [14-17]. Previous studies from Ethiopia indicate that Khat use is more common among Muslims compared to Christians [15], men and younger people [18]. Socio-cultural factors, in particular related to religion, are considered to be the most important drivers of khat use in Ethiopia, and help to explain the low use of khat in women [19]. In a previous study among people living with HIV (PLHIV) in Ethiopia, the prevalence of khat use was found to be 5.3% (n = 17) [20]. However, in a high risk group for HIV infection, female sex workers khat use was reported by nearly 80% [21]. In several studies, khat use has been found to be associated with higher levels of mental distress [18,22,23], although some Ethiopian studies have found no association [24].

Khat use has been suggested to be a risk factor for unprotected sex and HIV transmission; due to increased risk of multiple sexual partners and reduced condom use [21,25,26]. In an HIV testing and counseling center in Addis Ababa, Ethiopia, heavy users of khat were seven times more likely to have serum HIV positivity compared to non-users [27].

Although the prevalence of khat use and its physiological and psychosocial effects have been studied in diverse populations and areas of Ethiopia, there is little known about the prevalence of khat use in people living with HIV and factors associated with khat use in this group. This study aimed to assess the prevalence of khat use among people living with HIV who are receiving treatment in south west Ethiopia and examine the psychosocial and clinical factors associated with khat use in this population.

## Methods

### Study area

A cross-sectional study was conducted in Jimma University Specialized Hospital, which is located in the south west of Ethiopia. Jimma University Specialized Hospital (JUSH) is one of the oldest hospitals in the country and provides services for approximately 9,000 inpatient admissions and 80,000 outpatient attendances per year, serving a catchment population of about 15 million people [28,29]. In 2012, 6,561 PLHIV received services from the hospital: 3,524 were treated with antiretroviral therapy (ART) and 3,037 were under pre-ART follow-up. Data were collected from adult PLHIV who attended the JUSH HIV clinic for follow-up care during September 2012.

### Instruments

#### Dependent variable: khat use

A structured, interviewer-administered questionnaire was used to assess the pattern of khat use, including frequency. In this study, current khat use was defined as using (chewing) khat during the month prior to the interview (see Additional files 1 and 2).

#### Factors associated with khat use

A structured questionnaire was used to assess the socio-demographic characteristics and socio-economic status of participants (age, gender, marital status, education, occupational status, ethnicity, religious affiliation, frequency of attending a place of worship and living circumstances).

#### Clinical characteristics

Data regarding World Health Organisation (WHO) clinical staging of HIV status classification and CD4 count were extracted from patients’ medical records.

The Kessler 6-item scale (Kessler 6), which has been translated into Amharic and validated in Ethiopia [30], was used to measure mental distress (depressive, anxiety and somatic symptoms). The Kessler 6 was also translated into the Afan Oromo language for those participants who were unable to speak Amharic. Semantic validation was ensured. The Amharic version of the Kessler 6 has been demonstrated to have a sensitivity and specificity of 84.2% and 82.7%, respectively, at a cut-off point of 5 or more to screen for symptoms of common mental disorders [30].

The WHO’s Alcohol Use Disorder Identification Test (AUDIT) was used to measure alcohol use disorders (AUDs) [31]. A participant who scored eight or more on the AUDIT was classified as having an AUD. A self-report questionnaire was used to assess cigarette smoking (current smoker/non-smoker and the number and frequency of cigarettes smoked). Those participants who smoked at least one cigarette per day were considered to be cigarette smokers.

#### Medication adherence

In participants who were taking ART medication, self-report questions were used to assess whether the patient took ART drugs regularly or not (“Have you ever missed taking antiretroviral medications in the last month?”. If yes, “how often did you miss a dose?). The participant was asked the name of the medication and prescribed schedule, and then the number of times they had missed a dose of medication for each of the following time periods: ‘today’, ‘yesterday’, ‘in the past three days’, ‘in the past seven days’ and ‘in the past 30 days’.

All questionnaires were initially developed in English, translated into Amharic and Afan Oromo, and then back-translated to English to ensure semantic equivalence.

### Data collection procedures

Data were collected by seven health professionals with nursing qualifications (four of degree level and three with diploma) after three days of training on administration of the study instruments. Data collection was carried out after the questionnaires had been pretested on a sample (5% of the total sample) of people living with HIV attending the HIV clinic at Agaro health center, which is 45 km away from Jimma town. The data collection was supervised by a degree level health officer. The supervisor monitored data quality and checked all questionnaires for completeness.

### Sample size assumptions and sampling procedure

The sample size was calculated by assuming a prevalence of khat use of 50%, with any particular outcome to be with a 5% margin of error and 95% confidence interval of certainty (alpha = 0.05) and 10% non-response. Based on these assumptions, the sample size for the study was computed using a one-sample population proportion formula. The total required sample size was 401.

All eligible adult attendees of the ART clinic at JUSH during the study period were invited consecutively to participate in the study. However, patients with severe mental illness, those patients whose age was less than 18 years old and persons who were too physically unwell to participate in the study were excluded.

### Data analysis

After double data entry, data were exported from Epi-Data (version 3.1) [32] and analyzed using the Statistical Package for Social Sciences (SPSS, version 16). The dependent and independent variables were entered into a bivariate logistic regression analysis, one by one, in order to estimate the strength of association using Odds Ratios (OR). All variables associated with khat use in the bivariate logistic regression with a p-value of less than 0.25 were entered together into a multivariable logistic regression in order to control for confounding.

### Ethical considerations

Ethical clearance was obtained from the Ethical Review Board of Jimma University. Written informed consent was obtained from each of the participants prior to participation. Information obtained was kept confidential and anonymous during all stages of the study.

## Results

### Participant characteristics

A total of 401 PLHIV were approached for enrolment in the study. Of these, 389 (97.0%) agreed to participate and 12 (3.0%) refused (seven females and five males).

The majority (63.2%; n = 246) of participants were female. The mean age of participants was 35.5 ± SD 9.78 years, ranging from 18 to 70 years. The largest proportion of participants was aged between 25 and 34 years, accounting for 44.7% (n = 174) of the sample, followed by the 35 to 44 years age group which accounted for 30.0% (n = 117) of participants (see Table 1).

**Table 1:** **Socio-demographic characteristics of people with HIV attending Jimma University Specialized Hospital (n = 389)**

Characteristics		Frequency (%)
Sex	Male	143 (36.8)
	Female	246 (63.2)
Age (years)	18-24	23 (5.9)
	25-34	174 (44.7)
	35-44	117 (30.1)
	45-54	52 (13.4)
	55 and above	23 (5.9)
Ethnicity	Oromo	182 (46.8)
	Amhara	105 (27.0)
	Gurage	10 (2.6)
	Kefa	30 (7.7)
	Yem	17 (4.4)
	Others^1^	45 (11.6)
Religion	Orthodox Christian	200 (51.4)
	Muslim	125 (32.1)
	Protestant Christian	58 (14.9)
	Catholic	6 (1.5)
Education	Illiterate	71 (18.3)
	Read and write only	11 (2.8)
	Primary (1–8 grade)	172 (44.2)
	Secondary (9–12 grade)	102 (26.2)
	Tertiary (>12 grade)	33 (8.5)
Marital status	Single	48 (12.3)
	Married	193 (49.6)
	Divorced	92 (23.7)
	Separated	15 (3.9)
	Widowed	41 (10.5)
Occupation	Unemployed	97 (24.9)
	Daily laborer	99 (25.4)
	Employed	55 (14.1)
	Farmer	9 (2.3)
	Merchant	63 (16.2)
	Student	3 (0.8)
	Retired	11 (2.8)
	Other^2^	52 (13.4)
Living circumstances	Living alone	84 (21.6)
	Living with nuclear family	279 (71.7)
	Other^3^	26 (6.7)

^1^Other ethnicity = Dawuro, Wolayita or Tigray.

^2^Other occupation = Housewife, domestic worker or driver.

^3^Other living circumstances = Living with other relatives or non-relatives.

Out of the total participants, 85.3% (n = 332) were receiving ART and 14.7% (n = 57) were under pre-ART follow-up. Approximately half (46.8%; n = 182) of participants were Oromo in ethnicity while Orthodox Christians accounted for 51.4%, (n = 200) of the population. Nearly half of participants were married (49.6%, n = 193). Forty-four percent (n = 172) had achieved primary education. Around one quarter of participants were working as daily laborers (25.4%) and a further quarter were unemployed (24.9%) (see Table 1).

The overall prevalence of current khat use (in the last one month) among PLHIV was found to be 23.0% (n = 93). Among the people using khat, 39.8% (n = 37) reported that they used khat daily.

### Univariate associations with khat use

In univariate analyses, current khat use was associated significantly with male gender, being a follower of Islam (compared to Orthodox Christianity or Catholicism), current cigarette smoking, having an alcohol use disorder (scoring 8 or more on the AUDIT), high levels of common mental disorder symptoms (5 or more on the K6), having a CD4 count ≤ 200 cells/mm^3^ and missing doses of ART (Tables 2 and 3).

**Table 2:** **Socio-demographic factors associated with khat use among people with HIV attending services at Jimma University Specialized Hospital (n = 389)**

Variables		Khat use N (%)	Unadjusted odds ratio (OR)	95% confidence interval (CI)
Gender	Male	48 (33.6)	2.26	1.41, 3.63
	Female	45 (18.3)	Reference	
Age (years)	18-24	5 (21.7)	0.90	0.32, 2.58
	25-34	41 (23.6)	Reference	
	35-44	33 (28.2)	1.27	0.75, 2.17
	45-54	11 (21.2)	0.87	0.41, 2.85
	55 and above	3 (13.0)	0.49	0.14, 1.72
Ethnicity	Oromo	50 (27.5)	Reference	
	Amhara	24 (22.9)	0.78	0.45, 1.37
	Gurage/Keffa/Yem/Others	19 (18.6)	0.60	0.33, 1.41
Religion	Orthodox	33 (16.5)	0.30	0.18, 0.50
	Muslim	50 (40.0)	Reference	
	Catholic/Protestant	10 (15.6)	0.28	0.13, 0.60
Frequency of going worship	Daily/2–3 times weekly	37 (22.0)	1.95	0.88, 4.43
	Weekly	45 (23.7)	Reference	
	Less than once a week/Never	11 (35.5)	1.77	0.79, 3.98
Education	No formal education	20 (24.4)	0.83	0.46, 1.53
	Primary	48 (27.9)	Reference	
	Secondary	20 (19.6)	0.63	0.35, 1.14
	Tertiary	5 (15.2)	0.46	0.17, 1.26
Occupation	Unemployed	20 (20.6)	0.60	0.31, 1.15
	Daily laborer	30 (30.3)	Reference	
	Employed	14 (25.5)	0.79	0.37, 1.65
	Farmer	1 (11.1)	0.29	0.03, 2.40
	Merchant	14 (22.2)	0.66	0.32, 1.37
	Others^4^	14 (21.2)	0.62	0.30, 1.28
Marital status	Single	13 (27.1)	1.30	0.63, 2.67
	Married	43 (22.3)	Reference	
	Separated/Divorced/Widowed	37 (25.0)	1.16	0.70, 1.92
Living circumstances	Alone	25 (29.8)	1.58	0.91, 2.74
	With nuclear family	59 (21.1)	Reference	
	With others^5^	9 (34.6)	1.97	0.84, 4.65

^4^Occupation = Housewife, domestic worker, student, retired or driver.

^5^Living condition = living with other relatives or non-relatives.

**Table 3:** **Clinical and psychosocial factors associated with khat use among people with HIV attending services at Jimma University Specialized Hospital (n = 389)**

Characteristics		Khat use N (%)	Unadjusted odds ratio	95% confidence interval
Smoking 1 or more cigarettes per day	Yes	27 (75.0)	13.05	5.86, 29.05
	No	66 (18.7)	Reference	
Alcohol use disorder (scoring ≥8 on AUDIT scale)	Yes	42 (33.1)	2.04	1.27, 3.30
	No	51 (19.5)	Reference	
Mental distress (≥5 on Kessler 6 scale)	Yes	52 (29.5)	1.76	1.10, 2.82
	No	41 (19.2)	Reference	
CD4 count	≤200 cells/mm^3^	22 (37.3)	2.24	1.24, 4.04
	>200 cells/mm^3^	71 (21.5)	Reference	
Missing antiretroviral medication	Yes	10 (40.0)	2.39	1.03, 5.56
	No	67 (21.8%)	Reference	
WHO clinical stage	Stage 1	40 (22.5)	0.59	0.32, 1.11
	Stage 2	28 (29.9)	0.57	0.29, 1.12
	Stage 3	21 (32.8)	Reference	
	Stage 4	4 (21.1)	0.55	0.16, 1.85

### Factors associated with khat use in multivariable analysis

In the multivariable logistic regression model (See Table 4), lower khat use was found in Christians (Orthodox and Catholic): adjusted odds ratio (aOR) 0.26, 95% CI = 0.13, 0.55. Higher khat use was associated with current cigarette smoking (aOR 21.21, 95% = 7.19, 62.51), high levels of common mental disorder symptoms (aOR 1.84, 95% CI = 1.01, 3.36), alcohol use disorders (aOR 2.16, 95% CI = 1.10, 4.21), having CD4 count of ≤200 cells/mm^3^ (aOR 3.56, 95% CI 1.60, 7.50) and missing ART medication (aOR 4.20, 95% CI = 1.80, 5.75). In the final model, gender, age, ethnicity, frequency of attending a place of worship, education, occupation and living circumstances were not associated with khat use.

**Table 4:** **Multivariable logistic regression of factors associated with khat use among people with HIV attending services at Jimma University Specialized Hospital (n = 389)**

Characteristics		Adjusted odds ratio	95% confidence interval
Gender	Male	1.20	0.58, 2.50
	Female	Reference	
Age (years)	18-24	0.75	0.22, 2.57
	25-34	Reference	
	35-44	0.71	0.35, 1.44
	44-54	0.47	0.16, 1.37
	55 and above	0.15	0.03, 0.81
Religion	Christians (orthodox, catholic and protestant)	^.^0.26	0.13, 0.55
	Muslims	Reference	
Mental distress	Yes	1.84	1.01, 3.36
	No	Reference	
Smoking cigarettes	Yes	21.21	7.19, 62.51.
	No	Reference	
AUDs	Yes	2.16	1.10, 4.21
	No	Reference	
CD4	≤200 cell/mm^3^	3.46	1.60, 7.50
	>200 cell/mm^3^	Reference	
Missing ART drugs	Yes	4.20	1.80, 5.75
	No	Reference	

## Discussion

In this cross-sectional survey of PLHIV receiving care in a hospital in south west Ethiopia, the prevalence of current khat use was 23%. In our study, khat use was associated strongly with other substance misuse and with high scores on a measure of mental distress. Adverse clinical factors (poorer ART adherence and low CD4 count) were also associated with khat use in this population.

The prevalence of khat use found in this study was higher than that found in a previous study of PLHIV in south west Ethiopia (5.3%) [20]. This discrepancy might have arisen due to the difference between study populations. In the previous study from Jimma, PLHIV who were ART naïve were not included. Our study findings were comparable with findings from a study carried out in Gamo Gofa, south west Ethiopia, among youths attending for HIV testing and counseling (prevalence of khat use 27%) [33].

The prevalence of khat use found in this study was less than that measured in a community-based study carried out in Jimma town (30.8%) [16] and in a survey of Jimma University staff (30.6%) [17]. The lower prevalence of khat use in PLHIV in our study compared to these previous Jimma population studies might be related to HIV status, for example, PLHIV being less likely to use khat because they felt ill or they thought that khat using may have a negative impact on the potency of the antiretroviral drugs. Another possible reason for the lower reported prevalence could be higher social desirability bias in our study as our data collectors were health professionals and the face-to-face interviews were conducted within the ART clinic setting.

Khat use was more prevalent among male patients than female patients (33.6% vs.18.3%), which is consistent with the previous Ethiopian studies from Butajira, Jimma and Gamo Gofa [15,17,33]. This difference is likely to be due to the cultural restrictions on the use of substances, including khat, in women although a substantial percentage of women with HIV were using khat in this study (18.3%). Christians had 0.26 times lower odds of khat use than Muslims which is consistent with a community based study carried out in Butajira, Ethiopia [15].

### Khat use and mental health and other substance use problems

Khat use was more prevalent among PLHIV with alcohol use disorders (33%) compared to patients without AUDs (19.5%) which accords with study findings from Gamo Gofa, Ethiopia [33]. The reason might be that khat users drink alcohol in order to counteract the insomnia associated with khat [16].

In this study mental distress among khat users was more prevalent compared to patients who were not current khat users (29.5% vs.19.2%). The possible reason might be that patients with mental distress use khat in order to get relief from symptoms of mental distress; or khat use itself might lead to symptoms of anxiety and depression. However, it is difficult to establish the cause and effect relationship since our study design is a cross-sectional survey. A cross-sectional association between khat use and mental distress was also seen in previous community based studies from Ethiopia [14,18].

### Clinical factors and khat use

The prevalence of khat use among patients with CD4 > 200 cells/mm^3^ was 21.5% (n = 71). However, among patients with CD4 ≤ 200 cells/mm^3^ it was 37.0% (n = 22). This finding was unexpected and needs further investigation. One possible explanation is that khat users have poor adherence to ART regimens, which in turn may lead to reduced CD4 count. This explanation has some support from this present study. A more direct physiological effect of khat on CD4 count is also possible. Reverse causality is another possible explanation, if people with low CD4 counts use khat for relief of symptoms arising from HIV infection. The cross-sectional study design does not allow us to elucidate the direction of association.

The odds of missing ART medication among patients who use khat was nearly three times higher than the odds in non-khat users. From studies carried out on mice, khat has been shown to affect short-term memory adversely and so patients who are intoxicated with khat might be at increased risk of forgetting to take their ART mediation [34]. A qualitative study carried out in Ethiopia among people with schizophrenia found that many respondents were fearful that khat might interact with their medication, leading patients to choose one or the other [35]. However, patients and their caregivers also explained their ongoing khat use was necessary to improve the patient’s functioning and to alleviate medication side effects [19]. The extent to which these insights into the use of khat in people with schizophrenia pertain to people with HIV requires qualitative exploration.

### Potential limitations

Our measures of ART adherence were based on self-report responses from participants, which may not be sensitive to detect non-adherence. Additionally, social desirability bias may be an important limitation of the study as people who chew khat might minimize the frequency and amount of khat they use or they may refuse to disclose their khat use in an interview setting. The Afan Oromo version of the Kessler 6 has not been validated in Ethiopia which may limit interpretation of the cut-off to define ‘mental distress’; however, as the majority of respondents completed the Amharic version of the scale this may not be problematic. The study did not set out to examine socio-cultural factors associated with khat use and we did not explore reasons for starting khat use and the relationship with starting other substances. These are important areas for future study. Lastly, we used consecutive sampling which limits the generalisability of the study findings to all PLHIV.

## Conclusions

A significant proportion of people living with HIV in this study continued using khat while receiving ART medications. Furthermore, there was a positive association between khat use and missing doses of ART medication which might have serious consequences for the health and prognosis of these patients. Therefore, when working on ART adherence, it is important to also address khat use. There is a need to adapt and evaluate feasible and acceptable interventions to reduce khat use in people living with HIV. This study has also generated a hypothesis which requires testing about the effect of chronic khat use on CD4 counts in PLHIV.

## Additional files

Additional file 1:
**Structured questionnaire on khat use and use of other substances (English version).**

Additional file 2:
**Structured questionnaire on khat use and use of other substances (Amharic version).**
