# Supplementary material for: Khat use in people living with HIV: a facility-based cross-sectional survey from South West Ethiopia
Source: BMC Psychiatry. 2015 Apr 3;15:69. doi: 10.1186/s12888-015-0446-5 (PMC4394566; doi:10.1186/s12888-015-0446-5)
Supplement: Additional file 1: — Structured questionnaire on khat use and use of other substances (English version). [file 12888_2015_446_MOESM1_ESM.docx]

| 305 | Have you chewed khat in the past 30 days? | A. yes  B. No |
| --- | --- | --- |
| 306 | If yes, to question number 305, how often? | 1. Daily 2. 2-3 times per week 3. Once a week 4. Less than weekly |
| 307 | Do you smoke cigarettes? | 1. Yes 2. No |
| 308 | If yes to question number 307, how many cigarettes per day? | _____________________ |
| 309 | Do you use substance like hashish, ganja, and cannabis/marijuana? | A. yes  B. No |
| 310 | If yes to question number 309, how often? | 1. Daily 2. 2-3 times per week 3. Once a week 4. Less than weekly |

PART- III QUESTION ON KHAT AND OTHER SUBSTANCES USE

Part VI- Question regarding ART

| 601 | Have you ever missed taking your ART tablets in the last month? | - 1. Yes   2. No |
| --- | --- | --- |
| 602 | If your answer to Q 601 is yes, from the list of ART drugs, how often you miss taking them? | 1. Name of the drug ____________ 2. Should be taken____________times a day 3. Today you miss taking____________times 4. Yesterday you miss taking____________times 5. In the past 3 days you miss taking _______times 6. In the past 7 days you miss taking _______times 7. In the past 30 days you miss taking ______times |
