# Supplementary material for: Khat use in people living with HIV: a facility-based cross-sectional survey from South West Ethiopia
Source: BMC Psychiatry. 2015 Apr 3;15:69. doi: 10.1186/s12888-015-0446-5 (PMC4394566; doi:10.1186/s12888-015-0446-5)
Supplement: Additional file 2: — Structured questionnaire on khat use and use of other substances (Amharic version). [file 12888_2015_446_MOESM2_ESM.docx]

| 305 | ባለፈዉ 30ቀናት ዉስ*ጥ* ጫት ቅሞ ያቃሉ? | 1. አዎ 2. በፍፁም |
| --- | --- | --- |
| 306 | መልሶዎት አዎ ከሆነ ምን ያህል ጊዜ? | 1. በየቀኑ 2. በሳምንት ከ2-3 ጊዜ 3. በሳምንት አንድ ጊዜ 4. ከአንድ ሳምንት በታች 5. በፍጹም |
| 307 | ሲጋራ ያጪሳሉ ? | 1. አዎ 2. በፍፁም |
| 308 | መልሶዎት አዎ ከሆነ በቀን ምን ያህል ሲጋራ ያጪሳሉ? | _____________________ |
| 309 | አደንዛዥ ና አነቃቂ እጾችን ይጠቀማሉ ለምሳለ እንደ ማሪዋና /ሀሽሽ /ጋንጃ/ ካናቢስ የመሳሰለትን ? | 1. አዎ 2. በፍፁም |
| 310 | መልሶዎት አዎ ከሆነ ምን ያህል ጊዜ? | 1. በየቀኑ 2. በሳምንት ከ2-3 ጊዜ 3. በሳምንት አንድ ጊዜ 4. ከአንድ ሳምንት በታች 5. በፍጹም |

ክፍል III የጥያቄዎች

ክፍል VI - የፀረ ኤች አይ ቪ መድሀኒት የተመሇከተ ጥያቄዎች

| 601 | የፀረ ኤች አይቪ መድሀኒት አቋርጠዉ ያዉቃሉ? | 1. አዎ 2. የለም |
| --- | --- | --- |
| 602 | ለ ጥያቄ ቁ.601 መልስዎ አዎ ከሆነ ከተዘረዘሩት የፀረ ኤች አይቪ መድሀኒት ዉስጥ ምን ያህል ጊዜ መድሀኒት አልወሰዱም? | 1. የመድሀኒቱ ስም ____________ 2. ____________ ጊዜ በቀን የሚወሰድ 3. ዛሬ ____________ጊዜ መድሀኒትዎን ሳይወስዱ ቀርተዋል 4. ትናንት _______ጊዜ መድሀኒትዎን ሳይወስዱ ቀርተዋል 5. ባለፉት ሶስት ቀናት _______ጊዜ መድሀኒትዎን ሳይወስዱ ቀርተዋል 6. ባለፉት ሰባት ቀናት _______ጊዜ መድሀኒትዎን ሳይወስዱ ቀርተዋል 7. ባለፉት ሰላሳ ቀናት _______ጊዜ መድሀኒትዎን ሳይወስዱ ቀርተዋል |
